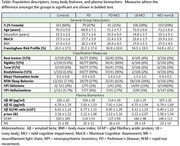# Alzheimer and Neurodegeneration Biomarkers in Mild Cognitive Impairment with Lewy Body Disease in COMPASS‐ND

**DOI:** 10.1002/alz70856_102379

**Published:** 2025-12-25

**Authors:** Richard Camicioli, Sandra E. Black, Michael J Borrie, Howard Chertkow, Jennifer G Cooper, Philippe Desmarais, Roger A. Dixon, Myrlene Gee, Ging‐Yuek Robin Hsiung, Zahinoor Ismail, Stephen Joza, Mario Masellis, Oury Monchi, Manuel Montero‐Odasso, Krista Nelles, Ronald B Postuma, Shady Rahayel, Eric E. Smith, Cheryl L Wellington, Canadian Consortium on Neurodegeneration (CCNA) and The Comprehensive Assessment of Neurodegeneration and Dementia Study (COMPASS‐ND) Investigators

**Affiliations:** ^1^ University of Alberta, Edmonton, AB, Canada; ^2^ LC Campbell Cognitive Neurology Research Unit, Sunnybrook Research Institute, University of Toronto, Toronto, ON, Canada; ^3^ Lawson Research Institute, London, ON, Canada; ^4^ Division of Aging, Rehabilitation and Geriatric Care, Lawson Health Research Institute, London, ON, Canada; ^5^ Baycrest and Rotman Research Institute, Toronto, ON, Canada; ^6^ University of British Columbia, Vancouver, BC, Canada; ^7^ Centre hospitalier de l'Université de Montréal, Montréal, QC, Canada; ^8^ Hotchkiss Brain Institute, University of Calgary, Calgary, AB, Canada; ^9^ Cognitive and Movement Disorders Clinic, Sunnybrook Health Sciences Center, Toronto, ON, Canada; ^10^ Research Centre, Institut universitaire de gériatrie de Montréal, Montreal, QC, Canada; ^11^ Schulich School of Medicine & Dentistry, Division of Geriatric Medicine, Western University, London, ON, Canada; ^12^ Montreal Neurological Institute, McGill University, Montreal, QC, Canada; ^13^ Centre for Advanced Research in Sleep Medicine, Hôpital du Sacré‐Cœur de Montréal, Montreal, QC, Canada; ^14^ Department of Clinical Neurosciences, University of Calgary, Calgary, AB, Canada

## Abstract

**Background:**

Mild Cognitive Impairment (MCI) may be caused by mixed pathologies. Blood markers indicative of Alzheimer pathology or neurodegeneration have not been extensively explored in patients with MCI who have features of Lewy Body disease (LB‐MCI) (cognitive fluctuations, parkinsonism, hallucinations, and REM‐sleep behavior disorder (RBD)). We compared plasma levels of amyloid beta (Aß), tau, glial‐fibrillary acidic protein (GFAP), and neurofilament light chain (NfL) between participants in COMPASS‐ND who met criteria for LB‐MCI with participant with MCI without these features, healthy controls (HC), and participants with Parkinson's disease (PD) with and without MCI.

**Method:**

Participants with MCI, HC, and PD were recruited as part of the COMPASS‐ND study and underwent assessment of demographic and clinical features. Participants with MCI were classified as LB‐MCI based on one or more criteria for LB disease. Plasma biomarkers were determined for amyloid species (Aß 42/40 ratio), tau‐181, GFAP, and (NfL using Simoa. Groups were compared using ANOVA with post hoc comparisons. Age and sex adjustment was done in ANCOVA models.

**Result:**

Among participants with MCI in COMPASS‐ND, there were 159 (97 M/62 F) with MCI but without LB features and 105 (46 M/32 F) with one or more of the LB‐MCI criteria. There were 161 HC (54 M/107 F), 79 PD (42 M/37 F), and 41 PD‐MCI (35 M/7 F). The Aß 42/40 ratio and tau‐181 differed between groups, with the LB‐MCI group showing a lower Aß 42/40 ratio and higher tau‐181 than the other groups. Post hoc comparison indicated that the ratio was lower in LB‐MCI than HC, PD, and PD‐MCI while tau‐181 was higher than HC, PD, and MCI without LB features. GFAP and NfL did not differ across groups. Age and sex adjustment did not alter the main findings.

**Conclusion:**

In COMPASS‐ND, participants with features of LB‐MCI showed a biomarker profile suggestive of high Alzheimer co‐pathology. Higher tau‐181 suggests that these individuals might have a higher pathological burden than the other groups. The influence of other co‐pathologies (i.e., vascular) and the influence on outcomes will be examined in this cohort. Future studies should examine for the presence of synuclein pathology across these groups.